# Using Cognitive Task Analysis to train Orthopaedic Surgeons - Is it time to think differently? A systematic review.

**DOI:** 10.1016/j.amsu.2020.09.031

**Published:** 2020-09-23

**Authors:** Karam Ahmad, Rahul Bhattacharyya, Chinmay Gupte

**Affiliations:** MSk Lab, Imperial College London, 2nd Floor, Sir Michael Uren Hub, 86 Wood Lane, London W12 0BZ, UK

**Keywords:** Orthopaedic surgery, Simulation, Cognitive task analysis, Training, Surgical education

## Abstract

****Background**:**

Working time restraints; senior led care; and a reduction in ‘out of hours’ operating has resulted in less operating time for orthopaedic trainees in the United Kingdom. Therefore, there has been an attempt to overcome these challenges by implementing novel techniques. Cognitive Task Analysis (CTA) focuses on the mental steps required to complete complex procedures. It has been used in training athletes and in general surgery but is new to orthopaedic training.

****Aim**:**

To undertake a systematic review to analyse if CTA is beneficial to train novice surgeons in common orthopaedic and trauma procedures.

****Materials and methods**:**

A systematic review was performed evaluating CTA in trauma and orthopaedic surgery on MEDLINE and EMBASE. Search terms used were: 'Cognitive task’, ‘mental rehearsal’ and ‘Orthop*'']. 33 studies were originally identified. Duplicate studies were excluded (11). Articles not relating to Orthopaedic surgery were excluded (15). The CTA research ranking scale was used to evaluate the impact of the studies included.

****Results**:**

7 studies were identified as appropriate for inclusion. 264 participants. 178 M, 86F. All studies showed objective or subjective benefits from CTA in orthopaedic training when compared to traditional methods. The majority of the participants highlighted high subjective satisfaction with the use of the CTA tools and reported that they proved to be excellent adjuncts to the traditional apprenticeship model.

****Conclusion**:**

CTA learning tools have demonstrated significant objective and subjective benefits in trauma and orthopaedic training. It is cost effective, easily accessible and allows repeated practice which is key in simulation training.

## Highlights

• Changes to training have resulted in less theatre time for Orthopaedic trainees.
• Cognitive Task Analysis (CTA) focuses on understanding technical steps and cognitive decision making required for procedures.
• It reduces the initial steep phase of the learning curve for trainees.
• This improves efficiency of training in the operating theatre.
• It has been shown to be beneficial in training Orthopaedic surgeons.

## 1 Introduction

Despite changes to orthopaedic training it is well established that current trainees have significantly less theatre training time as compared to their predecessors [1]. The shift from time-based to competency-based training has worsened the current situation [1]. In an attempt to counter both a reduction in theatre time for trainees and a rising demand for skilled surgeons, the development of simulation as an adjunct to the apprenticeship system has aided trainees to achieve their required training needs.

Simulation is ‘a method or technique that is employed to produce an experience without going through the real event’ [2]. It occurs in a safe environment and has been shown to improve confidence for surgeons [3]. It is heavily advocated in other specialities such as Emergency Medicine [4], General Surgery [5] and by the Royal Colleges of Surgeons [6].

Cadaveric and virtual reality simulation has found increasing application within orthopaedic training [[7], [8], [9], [10]]. However, they are expensive and not readily accessible. Karam et al. have shown that in the 10.13039/100011408USA, 25% of training programs do not have a dedicated simulation facility and this is due to lack of sufficient funds [11]. The situation is no different in the UK [12]. In this setting, it is important to have a suitable adjunct to the traditional apprenticeship model.

Cognitive Task Analysis (CTA), which originates from the military, is a modern approach to simulation, placing emphasis on decision making and the thought processes behind each step of key procedures [13]. It allows trainees to learn ‘how to do’ a technical step, ‘why’ they are doing the step and any potential errors (and solutions) that they can make in each phase of a surgical procedure. Studies have highlighted that mastering surgery requires a high cognitive and mental ability [14]. Fitts and Posners's model for mastery of skill implies that cognitive staging is the primary mental process needed for learning, followed by associative and automated processes [15]. Skill acquisition by cognitive methods has been shown to produce changes in the brain which engage the primary motor complex [16].

A systematic review evaluating CTA in general surgery (2013) has given an insight towards how CTA can improve surgical performance [13] and in recent years CTA has been increasingly used in trauma and elective orthopaedic training [[17], [18], [19]].

The aim of this study is to evaluate whether CTA learning tools are of benefit to trainees in Trauma and Orthopaedic Surgery.

## 2 Materials and methods

### 2.1 Literature search strategy

**Fig. 1:**
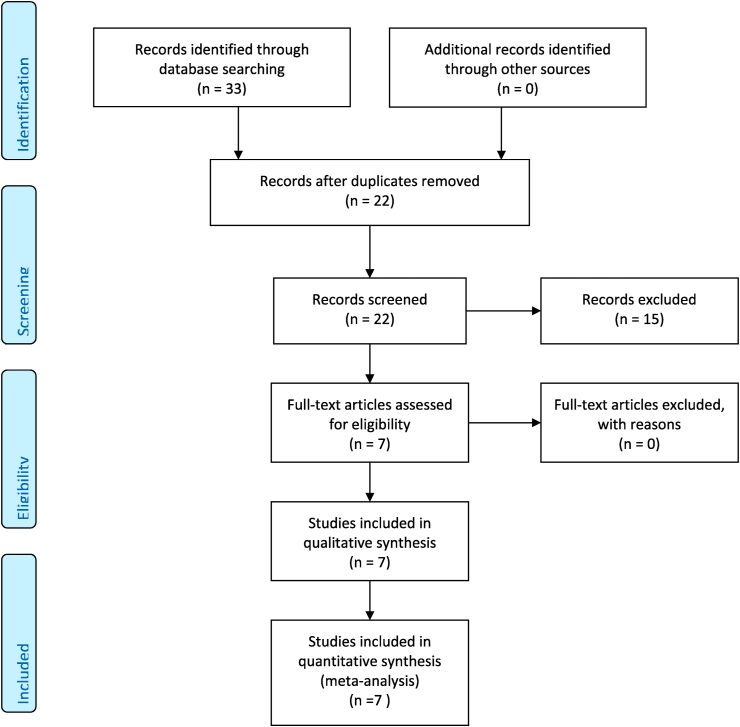
Prisma flowchart.

We reviewed studies on CTA in Orthopaedics on the Ovid MEDLINE and EMBASE databases. A guide stating the research question, search strategy, inclusion/exclusion criteria and risk of bias was formulated. The search and screening were performed by two of the authors, and disagreements were resolved by consensus. An electronic search was performed on November 17, 2019. No date limitations were placed. Only English articles were selected. Key words and phrases used were: ‘Cognitive task’ OR ‘mental rehearsal’ AND ‘Orthop*‘. Due to the limited published data on CTA all study types and trainee ranges were considered. The studies retrieved from the search were manually reviewed to identify other studies which could be relevant. Also reviewed were reference lists of included studies, study registries, and grey literature. This review has been reported in line with PRISMA (Preferred Reporting Items for Systematic Reviews and Meta-Analyses) (Fig. 1). And AMSTAR-2 (Assessing the methodological quality of systematic reviews) Guidelines.

### 2.2 Selection, inclusion and exclusion criteria

Studies which specifically analysed CTA in Orthopaedic surgery were evaluated. We defined CTA as a method by which knowledge for a procedure can be imparted via step by step protocols. 33 studies were identified. De-duplication reduced this to 22. Articles not relating to Orthopaedic surgery were excluded (15). Following inclusion/exclusion criteria, seven published articles were considered (Fig. 1). Demographics of the seven studies are shown in Table 1.

**Table 1:** Study demographics.

Author	Title	Year	No. of Participants	Level	Journal	Impact Factor (2018)	Study Type	Funding Source
Amer et al.	A Mobile-Based Surgical Simulation Application: A Comparative Analysis of Efficacy Using a Carpal Tunnel Release Module	2017	100	Medical Students	J Hand Surg Am	2.090	RCT	Internal
Bhattacharyya et al.	Knee Arthroscopy Simulation A Randomized Controlled Trial Evaluating the Effectiveness of the Imperial Knee Arthroscopy Cognitive Task Analysis (IKACTA) Tool	2017	16	Novice Orthopaedic Trainees	The Journal of Bone and Joint Surgery	4.716	RCT	Internal
Bhattacharyya et al.	Trauma simulation training: a randomized controlled trial evaluating the effectiveness of the Imperial Femoral Intramedullary Nailing Cognitive Task Analysis (IFINCTA) tool	2018	22	Medical Students	Acta Orthopaedica	3.076	RCT	AO Foundation, Switzerland, “Multipurpose Virtual Surgical Simulator”.
Levin et al.	Pre-course cognitive training using a smartphone application in orthopaedic intern surgical skills “boot camps”	2018	14	Orthopaedic Interns	Journal of Orthopaedics	1.907	Cohort	Internal
Logishetty et al.	A Multicenter Randomized Controlled Trial Evaluating the Effectiveness of Cognitive Training for Anterior Approach Total Hip Arthroplasty	2019	36	Surgical Residents- Post graduate year 1–4	The Journal of Bone and Joint Surgery	4.716	RCT	Royal College of Surgeons of England, United Kingdom CW1 Charity, United Kingdom, Johnson & Johnson, Switzerland.
Sugand et al.	Training effect of using Touch Surgery™ for intramedullary femoral nailing	2015	27	Medical Students	Injury Journal	1.834	Case Control	Internal
Sugand et al.	Validating Touch Surgery™: A cognitive task simulation and rehearsal app for intramedullary femoral nailing	2015	39 + 10	Medical Students and Orthopaedic Trainees	Injury Journal	1.834	Cohort	Internal

### 2.3 Scoring of CTA studies

A 5-point scoring system to appraise CTA was developed by Wingfield et al. in 2015 [13]. ‘The CTA Research Ranking Scale’ assesses journal impact score, study types and number of participants. This was used to assess selected studies prior to further in-depth analysis. Table .2 gives an overview of ‘The CTA Research Ranking Scale’.

**Table 2:** CTA research ranking scale.

**Journal Impact Score (2018)**	**Designated Point Value (1–5)**
<1.5	1
1.5–2.5	2
2.5–3.5	3
3.5–4.5	4
>4.5	5
	
**Study Type**	**Designated Point Value (1–5)**
Meta-Analysis	5
RCT	4
Cohort Study	3
Case-Control/Cross Sectional	2
Literature review	1
	
**Number of Participants**	**Designated Point Value (1–5)**
≥500	5
100–499	4
50–99	3
10–49	2
≤9	1

**Table 3:** Assessment type and associated results.

Author	Year	Title	CTA Method	Type of Assessment	Results	CTA Improved training?	Positive Subjective Outcome?	CTA Research Ranking
Amer et al.	2017	A Mobile-Based Surgical Simulation Application: A Comparative Analysis of Efficacy Using a Carpal Tunnel Release Module	Carpal Tunnel Surgery steps on ‘Touch Surgery™' vs Traditional Methods.	1. 21 question post-study standardised test	1. Test group average - 89.3% (±6.0%). Control group average 75.6% (±8.7%)	Yes	Yes	10
				2. Likert Scale for subjective rating	2. Overall content validity, quality of graphics, ease of use, and usefulness to surgery preparation rated as very high (4.8 of 5)			
Bhattacharyya et al.	2017	Knee Arthroscopy Simulation A Randomized Controlled Trial Evaluating the Effectiveness of the Imperial Knee Arthroscopy Cognitive Task Analysis (IKACTA) Tool	Imperial Knee Arthroscopy Cognitive Task Analysis (IKACTA) tool used to describe each phase of a diagnostic knee arthroscopy vs No additional learning material.	1. Validated Arthroscopic Surgical Skill Evaluation Tool [ASSET] global rating scale.	1. Mean ASSET score (and standard deviation) →IKACTA group = 19.5 ± 3.7 points. Control Group = 10.6 ± 2.3	Yes	Yes	11
				2. Likert Scale for subjective rating	2. All participants agreed that the cognitive task analysis learning tool was a useful training adjunct to learning in the operating room.			
Bhattacharyya et al.	2018	Trauma simulation training: a randomized controlled trial evaluating the effectiveness of the Imperial Femoral Intramedullary Nailing Cognitive Task Analysis (IFINCTA) tool	Imperial Femoral Intramedullary Nailing Cognitive Task Analysis (IFINCTA) tool used to describe each phase of antegrade femoral intramedullary nailing vs Standard operative technique manual.	1. Validated “Touch Surgery™” application assessment tool on femoral intramedullary nailing.	1. Post-test Median Score improvement (Intervention group over control group): Patient positioning and preparation- 20%, Femoral Preparation- 21%, Proximal locking- 10% and Distal Locking- 19%	Yes	Yes	9
				2. Likert Scale for subjective rating	2. All participants agreed the tool made the procedure easy to understand. The multi-modality approach was beneficial and that it was beneficial to use the tool prior to operating. 10/11 participants agreed that the tool was easy to use and 9/11 enjoyed using the tool.			
Logishetty et al.	2019	A Multicentre Randomized Controlled Trial Evaluating the Effectiveness of Cognitive Training for Anterior Approach Total Hip Arthroplasty	Anterior approach Total Hip Arthroplasty- Participants cognitively trained vs Training with a standard operation manual with surgical video.	1. Assessment of time taken, errors made, prompts required and acetabular cup orientation.	1. Cognitive trained- 35% faster, 69% fewer errors in instrument selection, 92% fewer prompts, Reduced inclination and anteversion errors.	Yes	Yes	11
				2. Training survey assessing the usability and applicability of the Cognitive Training Tool for Learning Total Hip Replacement.	2. 34 of 35 residents agreed that the CTT was useful for understanding technical skills, decision making, and common errors related to AA-THA, was easy and enjoyable to use, and contributed to a marked improvement over standard preoperative preparation			
Levin et al.	2018	Pre-course cognitive training using a smartphone application in orthopaedic intern surgical skills “boot camps”	Ankle open reduction + internal fixation and lag screw fixation using ‘Touch Surgery™'	1. Feedback from participants via post course survey	1. 10/14 participants believed using CTA improved baseline understanding, 9/14 believe learning was accelerated and 8/14 felt the application made the procedure easier to learn.	Yes	Yes	7
Sugand et al.	2015	Training effect of using Touch Surgery™ for intramedullary femoral nailing	Intra-medullary femoral nailing on ‘Touch Surgery™'- Consisting of four modules, participants completed a pre-module MCQ, had 6 attempts at each module and then completed a post module MCQ.	1. Pre- and Post- module MCQs, Comparison of Post module MCQ with Experts	1. Module- (i) patient positioning and preparation- 83% improvement (ii) femoral canal preparation- 94% improvement (iii) proximal locking 90% improvement (iv) distal locking and closure- 89% improvement. P- Value < 0.001 Similar post module scores between novices and experts.	Yes	N/A	6
Sugand et al.	2015	Validating Touch Surgery™: A cognitive task simulation and rehearsal app for intramedullary femoral nailing	Intra-medullary femoral nailing on ‘Touch Surgery™'- Consisting of four modules, done by experts and novices.	1. Construct validity using objective metrics.	1. Experts outperformed novices to demonstrate construct validity. Module - (i) Patient positioning and preparation- 32.5% higher, (ii) Femoral canal preparation- 31.5% higher, (iii) Proximal locking- 22.5% higher, (iv) Distal Locking and closure - 17% higher	Yes	Yes	7
				2. Face and content validity using a subjective questionnaire.	2. Both cohorts rated the face validity, quality of graphics, willingness to use the app, usefulness for preoperative rehearsal as good or very good. Experts rated the content validity as good too.			

## 3 Results

### 3.1 Participants

264 participants across 7 studies. 178 Male and 86 Female. There was an average of 37.7(Range:14–100) participants per study. Expertise ranged from medical students to senior orthopaedic trainees and consultants.

### 3.2 Summary of cognitive task analysis methodology and learning principles used (Table 3)

Amer et al. [20] randomized 100 medical students to learn carpal tunnel release surgery via video lectures or by using the Touch Surgery™ [20] application. Those who had observed or participated in Carpal tunnel release and used the app previously were excluded. The control group watched the presentation three times in one sitting. The intervention group completed the ‘Carpal Tunnel Surgery’ module three times on the app. The same standardised test of 21 multiple-choice questions was completed. The intervention group was asked to rate the app using a 5-point Likert rating scale (ranging from very poor to very good).

Bhattacharyya et al. [17] undertook a randomized controlled trial to evaluate the effectiveness of the Imperial Femoral Intramedullary Nailing Cognitive Task Analysis (IFINCTA) tool. A modified-Delphi technique was used to design a combined written and audio-visual tool in femoral intramedullary nailing. 22 medical students were randomized in two equal groups. The intervention group were taught using the CTA tool and the control group were given a standard operative manual. The students were scored on MCQs and how effectively manual steps were completed using finger swipes on the smartscreen.

Bhattacharyya et al. [18] carried out a randomized control trial to evaluate the effectiveness of CTA in knee arthroscopy. The cognitive task tool was developed and designed using the modified-Delphi method. It utilized written, visual video and audiological information simultaneously to train novices in this procedure. A double blind RCT was then undertaken to analyse its efficacy to train novices in diagnostic knee arthroscopy. An objective assessment using the Validated Arthroscopic Surgical Skill Evaluation Tool [ASSET] global rating scale was used and a subjective assessment to evaluate participant satisfaction was undertaken utilizing a Likert rating scale.

Levin et al. [21] had 14 Orthopaedic interns who evaluated the Touch Surgery app: interns completed a simulated ankle open-reduction, internal-fixation and lag screw fixation prior to attending an annual four-week bootcamp. Participants completed the learning module, and then multiple-choice questions (Pass-mark 70%). They were required to pass the exam and allowed multiple attempts if required. A post-course survey was provided to participants on completion.

Logishetty et al. [19] developed procedural steps for Anterior Approach Total Hip Arthroplasty (AA-THA) via a modified-Delphi technique using 4 expert arthroplasty surgeons. 36 surgical residents were block randomized in two equal groups (residents who had previously observed or performed AA-THA were excluded). The intervention group were given online access to the cognitive tool and the control group were given a standard operation manual for this procedure.

Sugand et al. [22] randomized 27 medical students to evaluate the training effect of the touch surgery application in femoral intramedullary nailing. The students completed a pre-module questionnaire, a test module, of which they had six attempts each and a post-module MCQ. Scores were given for decision making, swipe interactions, and time taken to complete steps. Percentage total scores were calculated.

Sugand et al. [23] attempted to validate Touch Surgery™ for Intramedullary Femoral Nailing (IFN). As per Sugand's previous study [22], the procedure was divided into four modules. Real-time objective performance data was obtained and stored from the participants primary attempt. This was used to assess construct validity. A post-study questionnaire using the Likert scale was used to assess face and content validity.

### 3.3 Assessment and study results (Table 3)

All studies found objective or subjective benefits in using CTA.

Objectively, in the study by Amer et al. [20], the intervention group scored on average 89.3% (±6.0%) compared to 75.6% (±8.7%) on the 21 question post-study standardised test.

Both studies by Bhattacharyya et al. [17,18] found that the intervention group scored higher than the control on CTA's for f for both knee arthroscopy and femoral nailing. For the arthroscopic Knee CTA, the ASSET score was on average 19.5 ± 3.7 for the intervention group, and 10.6 ± 2.3 for the control. For the INFINCTA tool, intervention group participants reported median post test score improvements of 20% in Patient positioning and preparation, 21% in Femoral Preparation, 10% in Proximal locking and 19% in Distal Locking (in comparison to control group participants).

The study on CTA and AA-THA by Logishetty at al [19] found cognitively trained participants were on average 35% faster, made 69% fewer errors in instrument selection, and required 92% fewer prompts. They also were more accurate with acetabular cup orientation.

Both studies by Sugand et al. [22,23] showed benefits of CTA based simulation for intramedullary femoral nailing. On assessing the effect of CTA on training [22], Sugand found that novice participants improved (following CTA based simulation) by 83% in Patient positioning and preparation, by 94% in Femoral canal preparation, by 90% in Proximal locking 90% and 89% in Distal locking and closure. To demonstrate construct validity [23] experts outperformed novices in each module by 32.5% in Patient positioning and preparation, 31.5% in Femoral canal preparation, by 22.5% in Proximal locking and by 17% Distal Locking and closure. Levin et al. [21] did not contain any objective measure of CTA.

Subjectively, participants from Amer et al. [20] rated content validity, quality of graphics, ease of use, and usefulness to surgery preparation as very high. Bhattacharyya et al. [17,18], found that participants agreed the cognitive task analysis learning tool was a useful training adjunct to learning in the operating room. Over 90% of participants found to tool easy to use and enjoyed using it. Levin et al. [21] found that 10/14 participants believed using CTA improved baseline understanding, 9/14 believed learning was accelerated and 8/14 felt the procedure was easier to learn as a result of this.

All participants in the AA-THA study by Logishetty et al. [19] found the CTA tool useful to understand key technical steps, decision making processes, highlighting errors, and easy to use. 34/35 enjoyed using the tool.

When validating Touch Surgery™ for intramedullary femoral nailing, Sugand et al. [23] found that both junior and senior cohorts rated the face validity, quality of graphics, willingness to use the app, usefulness for preoperative rehearsal as good or very good. Experts also rated the content validity as good.

## 4 Discussion

The use of CTA within orthopaedic training is relatively novel. 6/6 studies show objective benefits when using CTA (one study did not use objective assessment). They suggest CTA enhances performance and efficiency in orthopaedic training. In the randomized controlled trials by Bhattacharyya et al. [17,18] and Logishetty et al. [19] expert created CTA tools (using the Delphi model) have been shown to clearly improve participants ability to complete procedures in trial scenarios. The creation of CTA tools for individual procedures can open the door to multi-centre collaborations for other procedures and potential inclusion in to training programs. Studies by Sugand et al. [22,23], and Amer et al. [20] support objective and subjective benefits in CTA based simulation training.

Using CTA the trainee is able to work in a safe, protected environment, with minimal restrictions and at a time and place which suits them. It is inexpensive, web-based and accessible that allows repetitive practice which is the cornerstone of simulation training. Trainees can progress faster through the initial phase of the Sigmoid learning curve [24]. This, a concept of mathematical psychology, follows the learner from unfamiliarity to mastery of a skill. Initially progress is slow, however with intentional practice, traction is gained, and one enters the stage of ‘hypergrowth’ (where learning is exponential) and then subsequent mastery [[22], [23]]. The aim of CTA is to propel the novice learner into hypergrowth prior to getting sustained theatre experience, thereby improving efficiency of their operating theatre training time. Furthermore, this enhances patient safety as trainees are more equipped with knowledge on the technical skills and potential errors before they perform a procedure for the first time on patients [17,19].

By undertaking CTA procedures, the trainee is enabled to progress through unconscious incompetence, conscious incompetence and potentially reach the stage of conscious competence. They, therefore, enter the learning process on patients at a higher point on the Broadwell learning curve [25].

Studies have estimated that 70% of vital steps can be missed out when taught by experts [13]. This is partially attributed to expert surgeons being in the unconscious competence stage of teaching. CTA provides an opportunity to rectify this flaw in traditional methods of training by being thorough and systematic.

In the current unprecedented situation due to the COVID-19 pandemic, CTA based simulation may find an increasing role in standardised orthopaedic training. This can be practiced remotely, repeatedly, with no human contact. The encouraging results of this systematic review can pave the way for future CTA based learning tools in other areas of orthopaedics, eventually leading to formal CTA learning programs for orthopaedic trainees worldwide.

### 4.1 Limitations

All studies reported thus far have not analysed transfer validity. It is important to evaluate whether the beneficial results viewed in the simulation setting translates to patients in the operating theatre.

There may be a role for the assessment of soft skills such as communication and leadership to be integrated into CTA.

As CTA based simulation is a developing topic there are a limited number of studies available to include in this review. Secondary to this, there is no clear outcome measure to evaluate CTA use between different procedures. Variance in expertise between participants can make comparison between CTA based training and conventional methods difficult. The positive findings of all studies mean publication bias must be considered as a potential limitation. Finally, registration of the work was completed on Research Registry which is a smaller sized registry compared to Prospero.

Despite limitations, CTA is a simulation tool which helps the novice learner develop a standardised level of competence in a procedure prior to doing it for the first time on a patient. In healthcare systems around the world today, the availability of such a tool is highly desirable.

### 4.2 Future work

Future work should focus on transfer validity of CTA to the operating room, creation of CTA based training tools for more procedures, and development of a clear, objective outcome measure. Once established, the incorporation of CTA into formal teaching curriculums would attempt to resolve some of the limitations of surgical training.

## 5 Conclusion

The current attempts to use CTA and other simulation methods, are secondary to strained health services, time pressures and new legislation reducing the time orthopaedic trainees can spend in the operating theatre. The innovation of CTA in Orthopaedics is a systematic, cost effective and easily accessible method of training that allows us to tackle the challenges faced by our future surgeons.

## Provenance and peer review

Not commissioned, externally peer reviewed.

## Ethical approval

No ethical approval was required.

## Funding

No funding was required.

## Consent

None.

## Registration of research studies

1. Name of the registry: Research Registry
2. Unique Identifying number or registration ID: reviewregistry948
3. Hyperlink to your specific registration (must be publicly accessible and will be checked): https://www.researchregistry.com/browse-the-registry#registryofsystematicreviewsmeta-analyses/registryofsystematicreviewsmeta-analysesdetails/5f1333e1daa3c40015766b01/

## Guarantor

Karam Ahmad, Rahul Bhattacharyya and Chinmay Gupte.

## CRediT authorship contribution statement

**Karam Ahmad:** Conceptualization, Writing - original draft, Conception, design, review of literature, drafting and final approval of the paper. **Rahul Bhattacharyya:** Conceptualization, Writing - original draft, Conception, design, review of literature, drafting and final approval of the paper. **Chinmay Gupte:** Conceptualization, Conception, design and final approval of the paper.

## Declaration of competing interest

None.
